# Divergent Responses of Hydrophilic CdSe and CdSe@CdS Core–Shell Nanocrystals in Apoptosis and In Vitro Cancer Cell Imaging: A Comparative Analysis

**DOI:** 10.3390/jfb14090448

**Published:** 2023-09-01

**Authors:** Kishan Das, Neelima Bhatt, Ajith Manayil Parambil, Kajal Kumari, Raj Kumar, Kamla Rawat, Paulraj Rajamani, Himadri B. Bohidar, Ahmed Nadeem, Saravanan Muthupandian, Ramovatar Meena

**Affiliations:** 1 School of Physical Sciences, Jawaharlal Nehru University, New Delhi 110067, India; kishanbhu2010@gmail.com (K.D.); bohidarjnu@gmail.com (H.B.B.); 2 Shaheed Rajguru College of Applied Sciences for Women, University of Delhi, New Delhi 110096, India; 3 School of Environment Sciences, Jawaharlal Nehru University, New Delhi 110067, India; neelimabhatt070@gmail.com (N.B.); ajithmptnr@gmail.com (A.M.P.); rajloro@rediffmail.com (R.K.); paulrajr@mail.jnu.ac.in (P.R.); 4 A.I. Virtanen Institute for Molecular Sciences, University of Eastern Finland, 70211 Kuopio, Finland; biotech.kajal@gmail.com; 5 Department of Chemistry, Jamia Hamdard University, New Delhi 110062, India; kamlarawat@jamiahamdard.ac.in; 6 Department of Pharmacology and Toxicology, College of Pharmacy, King Saud University, Riyadh 11451, Saudi Arabia; anadeem@ksu.edu.sa; 7 AMR and Nanomedicine Laboratory, Department of Pharmacology, Saveetha Dental College and Hospitals, Saveetha Institute of Medical and Technical Sciences (SIMATS), Chennai 600077, India

**Keywords:** water-soluble nanocrystals, size-dependent cytotoxicity, fluorescent cell imaging, apoptosis

## Abstract

With their distinctive core–shell design, core–shell nanocrystals have drawn interest in catalysis, medicinal research, and nanotechnology. These nanocrystals have a variety of characteristics and possible uses. The application of core–shell nanocrystals offers significant potential in increasing diagnostic and therapeutic approaches for cancer research in apoptosis and in vitro cancer cell imaging. In the present study, we investigated the fluorescence behavior of hydrophilic CdSe (core-only) and CdSe@CdS (core–shell) nanocrystals (NCs) and their potential in cancer cell imaging. The addition of a CdS coating to CdSe NCs increased the fluorescence intensity tenfold. The successful fabrication of core–shell CdSe@CdS nanocrystals was proven by a larger particle size (evaluated via DLS and TEM) and their XRD pattern and surface morphology compared to CdSe (core-only) NCs. When these NCs were used for bioimaging in MCF-7 and HEK-293 cell lines, they demonstrated excellent cellular uptake due to higher fluorescence intensity within cancerous cells than normal cells. Comparative cytotoxicity studies revealed that CdSe NCs were more toxic to all three cell lines (HEK-293, MCF-7, and HeLa) than CdSe@CdS core–shell structures. Furthermore, a decrease in mitochondrial membrane potential and intracellular ROS production supported NCs inducing oxidative stress, which led to apoptosis via the mitochondria-mediated pathway. Increased cytochrome c levels, regulation of pro-apoptotic gene expression (e.g., p53, Bax), and down-regulation of Bcl-2 all suggested cellular apoptosis occurred via the intrinsic pathway. Significantly, at an equivalent dose of core–shell NCs, core-only NCs induced more oxidative stress, resulting in increased apoptosis. These findings shed light on the role of a CdS surface coating in reducing free radical release, decreasing cytotoxicity, and improving fluorescence, advancing the field of cell imaging.

## 1. Introduction

Nanocrystals (NCs) are nanoparticles with exceptional photophysical and photochemical properties. Due to their size-dependent optical and electronic properties originating from quantum confinement effects, interest in nanocrystal research has grown significantly [1,2,3,4]. Compared to fluorescent proteins and organic dyes, semiconductor nanocrystals are more fluorescent in terms of brightness and stable against photo-bleaching [5,6]. A new discipline focusing on nano-bioactivity, exclusively devoted to nanocrystals, has emerged due to recent advancements in the production and bioconjugation of nanocrystals. The ability to make nanocrystals water soluble and enable bioconjugation has made this development possible. Nevertheless, despite these advancements, difficulties with their surface chemistry have restricted the use of nanocrystals in biological applications [7,8]. Nonetheless, nanomaterials with remarkable surface modifications, made using the bioconjugation protocols, have established their worth in the last decade [9].

Any biological application of a nanomaterial requires a prior screening of its cellular uptake and cytotoxicity profile. These two signature parameters depend on the nanoparticle’s size and surface morphology. Derfus et al. have shown that the processing parameters conditioned the cytotoxicity of CdSe nanocrystals during synthesis [10]. This toxicity arose from the leaching of Cd^2+^ ions from the CdSe lattice over a period of time. Su et al. have evaluated the cytotoxicity of multi-layer core–shell NCs and shown that CdTe NCs were highly toxic to K562 and HEK-293T cell lines due to the leaching of Cd^2+^ [11]. The core–shell structures were relatively more biocompatible [12,13]. Delivery of these NCs from the circulation to the target cells is an important step that involves cellular uptake, receptor trafficking, and intracellular delivery. Hild et al. described the ideal features a nanoparticle must possess for biological applications and cellular imaging [14]. The cytotoxicity of CdSe and ZnS nanoparticles with surface modifications using different functional groups such as 3-mercaptropionic acid (3-MPA), silane, and polymers was assessed extensively by Kirchner et al. [15]. These studies suggested that nanoparticle aggregation and the release of Cd^2+^ ions played a crucial role in their cytotoxic effects. Chan et al. revealed that CdSe nanoparticles induced apoptotic biochemical changes, loss in mitochondrial membrane potential, and the release of cytochrome c in the IMR-32 human neuroblastoma cell lines [16]. However, these biochemical changes were not seen when the NCs were surface-coated with ZnS. The size-dependent activation of autophagy caused by NCs was studied by Sleverstov et al. [17]. The effects of surface chemistry and particle size on the cellular and cytotoxicity in murine macrophage cell lines were extensively studied by Cliff et al. [18].

Toxicity is the limiting factor for their broad range of applications. In brief, NCs’ toxicity may depend on different factors such as their composition, size, and the chemical nature of the capping material. Despite the increasing number of publications on NCs’ toxicity, the effort to obtain safe and biocompatible NCs is also increasing, so that they can be used safely for various biological applications [19,20]. Mainly, two reasons suggest that bare-core nanocrystal use is impractical. Firstly, the imperfect crystalline structure of the nanoparticles causes emission defects, mainly blinking [21,22,23]. Secondly, due to their large surface area to volume ratio, the cores are highly reactive and hence unstable, which makes them prone to photo-bleaching [24].

Interactions of NCs with intracellular components, the release of Cd^2+^ ions (degradation of core-only NCs), and the generation of free radicals are responsible for NCs’ cytotoxicity [10]. Studies have observed that the degradation of NCs, hence the release of Cd ions, occurs whenever they are exposed to an oxidative environment and even during synthesis and processing. The addition of a different shell can achieve a reduction in oxidation [25]. Free radical production is an important factor that contributes to NCs’ toxicity [26,27,28].

Increased photochemical stability and higher quantum yields have been achieved with capping core nanocrystals [29]. The choice of shell and coating are the two important aspects to consider when producing such nanocrystals. This study presents a comprehensive investigation into the fluorescence behavior of hydrophilic CdSe (core-only) and CdSe@CdS (core–shell) nanocrystals, shedding light on their utility in cancer cell imaging. The findings highlight the significant enhancements in fluorescence intensity achieved through CdS coating and demonstrate the different cytotoxicity and apoptotic pathways associated with the two types of nanocrystals. These insights contribute to advancing the cell imaging field, paving the way for developing more effective and targeted imaging agents for cancer diagnosis and therapy. Further, this study presents a novel finding by elucidating the intricate relationship between particle size variations, the specific cell lines employed for experimentation, and their profound influence on nanoclusters’ cellular absorption dynamics. Remarkably, our research showcases the unprecedented significance of even the subtlest disparities in particle size and the precise selection of cell lines on the intricate mechanisms governing the uptake of these nanoscale clusters by cells.

## 2. Materials and Methods

### 2.1. Materials

Cadmium oxide (CdO, 99.99%), octadecene (ODE, 90%), oleic acid (OA, 90%), trioctylphosphine (TOP, 93%), and selenium (Se, 99.99%) were purchased from Sigma Aldrich (St. Louis, MO, USA). 3-Mercaptopropionic acid (3-MPA) and sulfur powder (S, USP sublimed) were purchased from Fisher Scientific (Waltham, MA, USA).

### 2.2. Synthesis of CdSe Nanocrystals

The synthesis of the CdSe nanocrystals, which follows a kinetic growth process, is discussed in our previous studies [30,31,32]. We could successfully prepare these NCs with different sizes, as confirmed by UV–Vis, dynamic light scattering, and TEM data. The physical characteristics of these NCs are listed in Table 1.

### 2.3. Synthesis of CdSe@CdS Core–Shell NCs and Characterization

The well-known carboxy-amine coupling reaction protocol for synthesizing CdSe@CdS core–shell nanostructures was used with some modifications [33]. Briefly, a mixture of CdO (256 mg), OA (2.4 mL), and ODE (10 mL) was heated to a temperature of 290 °C in a 100 mL flask. When the temperature reached 280 °C, the reacting solution turned colorless due to the formation of cadmium oleate. The CdSe quantum dots were made from a selenium solution dissolved in 2 mmol of Se prepared in 0.472 g of TOP diluted with 1.37 g of ODE. This was rapidly injected into the reaction flask maintained at 290 °C. Then, the reaction temperature was reduced to 250 °C, and the structures were grown until the required size was achieved (the growth period after nucleation was the key parameter), which took less than 15 min. For the synthesis of the core–shell nanostructures, a solution of CdO (0.76 g), OA (6 mL), and ODE (25 mL) was heated to 290 °C till it turned colorless. Once it cooled to ≤100 °C, sulfur powder (0.16 g) was dissolved in it. This solution was then added drop-wise to the CdSe reaction vessel, immediately initiating the nanocrystals’ nucleation and saturation growth. After synthesis, the aliquots were isolated by precipitation in a 1:1 mixture of ethanol and butanol, which was then centrifuged, and the nanocrystals with slurry were taken out. This was mixed in 3 mL of hexane. We noticed an excess of ODE and OA adsorbed to the nanocrystals’ surfaces. So, these samples were washed at least eight times with a 1:1 mixture of ethanol and butanol before their characterization. After that, the hydrophobic oleic acid-capped NCs were transformed into hydrophilic 3-MPA-capped NCs by ligand exchange.

The average size and morphology of the NCs were obtained using JEOL 2100F TEM (Tokyo, Japan), operated at 200 kV. The surface charges were measured using an electrophoresis instrument (ZC-2000, Microtec, Funabashi, Japan). The hydrodynamic sizes were measured using dynamic light scattering (DLS) at a scattering angle of θ = 90° with a laser of wavelength 632.8 nm (PhotoCoR Instruments, Beltsville, MD, USA). The hydrodynamic radius, R_h_, can be calculated by the corresponding diffusion coefficient *D* through the Stokes–Einstein equation given below.

(1) $$D=\frac{k_{B}T}{\left(6\pi {\eta_{0}R}_{h}\right)}$$

X-ray diffraction (XRD) patterns were collected using an XRD Rigaku D/Max 2200 diffractometer (Rigaku, Tokyo, Japan) with Cu Ka radiation (λ = 1.54 Å) in the 2θ range from 10 to 60°. UV–Vis absorption data were obtained using the Agilent Cary 60 UV–Vis spectrophotometer (Agilent, Santa Clara, CA, USA). A Varian Cary Eclipse fluorescence spectrophotometer (Varian, Palo Alto, CA, USA) collected the steady-state photoluminescence (PL) spectra.

### 2.4. In Vitro Biocompatibility and Imaging

#### 2.4.1. Cytotoxicity Assay

The cytotoxicity of the NCs was evaluated using an MTT assay. The cell lines were obtained from NCCS, Pune, India. The comparative cytotoxicity of CdSe and CdSe@CdS nanocrystals on one normal cell line (human embryonic kidney, i.e., HEK-293) and two cancerous cell lines (the HeLa, i.e., a human epithelial-like portion-carcinoma and human breast adenocarcinoma, i.e., MCF-7) were evaluated at various concentrations (3, 6, 9, and 12 pM) of CdSe and CdSe@CdS nanocrystals using the method of Meena et al. [34]. All experiments were performed in triplicate. The cells were examined at 570 nm in an ELISA reader. The % cytotoxicity was calculated using the following formula [34].

(2) $$\%\;Cytotoxicity=\frac{Absorbance\;of\;treated\;sample}{Absorbance\;of\;Control\left(untreated\right)samples}\times 100$$

#### 2.4.2. Measurement of Intracellular Reactive Oxygen Species (ROS) Level

Intracellular ROS induced by CdSe and CdSe@CdS NCs were detected by using 2′,7′-Dichlorofluorescein-diacetate (DCFH-DA) staining following the protocols of Kermanizadeh et al. [35]. The fluorescence intensity was measured using 485 excitation and 520 nm emission filters using a fluorimeter (RF-5301 PC Shimadzu spectrofluorometer, Nakagyo-ku, Kyoto, Japan).

#### 2.4.3. Electron Paramagnetic Resonance (EPR) Spectroscopy

EPR measurements of free radicals were carried out in a Bruker EMX Micro X spectrometer according to the modified protocol of Kaur et al. [35]. The treated cells were suspended in 100 mM DMPO. Treated and untreated cells were loaded in sealed quartz capillary tubes to obtain spectra by transferring them to the EPR cavity. For each sample, the 2D spectrum was recorded using a Bruker e-Scan EPR. Spectrometer quantitation of the EPR spectra and baseline correction were performed using the Bruker WinEPR data processing software, Version: 921201 (Bruker, Billerica, MA, USA).

#### 2.4.4. Microscopic Analysis

To analyze the effects of NCs on cellular morphology, the protocols used by yang et al. were followed [36]. Treated and untreated cells were cut into ultrathin (70–80 nm) sections by an ultramicrotome (Leica Ultracut—UCT) and observed under a transmission electron microscope (JEOL-JEM-2100F) after staining with uranyl acetate.

#### 2.4.5. Cellular Uptake

HeLa cells were obtained from the National Center for Cell Science, Pune, India, and suitably cultured in appropriate conditions. The cells were treated with various concentrations (3, 6, 9, and 12 pM) of CdSe and CdSe@CdS NCs and incubated for 12 h at 37 °C in 5% CO_2_. After the proper treatment, the treated cells were observed under bright UV (405 nm) and blue (488 nm) field excitation using an Olympus Fluo View TM FV1000 (Olympus, Tokyo, Japan) laser scanning confocal microscope and the fluorescence intensity was measured.

#### 2.4.6. Analysis of Mitochondrial Membrane Potential (MMP)

To study the loss in the mitochondrial membrane potential, the MMP was measured using mitochondrial-membrane-permeable dye, i.e., JC-1 (Flouroprobe-5,5′,6,6′-tetrachloro-1,1′,3,3′-tetraethylbenzimidazol-carbocyanine iodide) dye. In brief, cells were seeded in a 6-well plate and treated with the same concentration (12 pM) of CdSe and CdSe@CdS NCs. After 12 h of treatment, the treated cells were washed with PBS, stained with 2 µg/mL of JC-1 dye, and incubated at 37 °C in the dark for 30 min. Then, the cells were washed with PBS, and images were captured with a Nikon Eclipse 90i Epi fluorescence upright microscope (Nikon, Tokyo, Japan) equipped with a Nikon DXM 1200 digital camera and viewed at 20× magnification. The quantitative measurement of the mitochondrial membrane potential was performed using a fluorimeter. At the end of the exposure, the cells were incubated with the JC-1 (10 μg/mL in PBS) for 20 min at 37 °C. After that, the cells were harvested and washed with phosphate buffer saline. The fluorescence intensity was measured at 530/590 nm.

#### 2.4.7. Western Blot Analysis for Protein Extraction

An equal number of cells were grown in a 60 mm plate and, after 80% of confluency, the cells were treated with 12 pM of CdSe and CdSe@CdS NCs for 12 h. At the end of the treatment, the cells were harvested by trypsinization and washed with ice-cold PBS. After that, a whole cell protein suspension was prepared in RIPA buffer containing 1× protease inhibitors (G-Bioscience, New Delhi, India). The protein content was measured using a Bradford assay [37]. The proteins were separated in 8% SDS-PAGE gel and transferred to a nitrocellulose membrane. The membrane was blocked using 5% BSA in PBS and probed with primary p53, Bax, bcl-2, and caspase-3 antibodies, followed by incubation with secondary antibody anti-mice Ig-G. Image analysis software (Thermo Fisher Scientific, Waltham, MA, USA) performed the densitometry analysis of the protein band, and *β*-actin was used as an internal control.

#### 2.4.8. Cytochrome C in the Cytosolic Fraction

For isolation of the cytosolic fraction, after 12 h of treatment cells were trypsinized and washed in PBS and resuspended into 500 μL of fractionation buffer (buffer HEPES (pH 7.4) 20 mM, KCI 10 mM, MgCl_2_ 2 mM, EDTA 1 mM, EGTA 1 mM, DTT 1 mM, protease inhibitors cocktail—50 μL/10 mL buffer) and incubated for 15 min on ice. Using pass cells, the suspension was passed through a 27-gauge needle (1 mL syringe) 10 to 15 times, then kept on ice for a further 20 min, and further centrifuged at 3000 rpm for 5 min. The supernatant was collected and again centrifuged at 8000 rpm for 5 min. The pellet was discarded, and the supernatant was used to analyze the expression of cytochrome c in the cytosolic fraction. As mentioned in the protocol above, the extracted protein was separated in 8% SDS-PAGE gel for western blotting analysis.

#### 2.4.9. FACS Analysis

From the 60 mm culture dish, media was added into 6-well plates, which were then seeded with HeLa cell lines and allowed to grow overnight. NCs were then added to the cultured cells and allowed to interact for 24 h. After overnight treatment, cells were harvested and centrifuged at 400× *g* for 10 min. The obtained cell pellet was fixed with 70% ethanol and, after that, washed with PBS. FACS cells were stained with Annexin V FITC/PI as per the manufacturer’s instructions (Cayman Chemical Company, Ann Arbor, MI, USA). Then, the samples were analyzed with a flow cytometer (Beckman Coulter, Miami, FL, USA). At least 10,000 cells were analyzed to determine the percentage of apoptotic cells.

## 3. Results

### 3.1. Characterization of Nanoparticles

**Figure 1:**
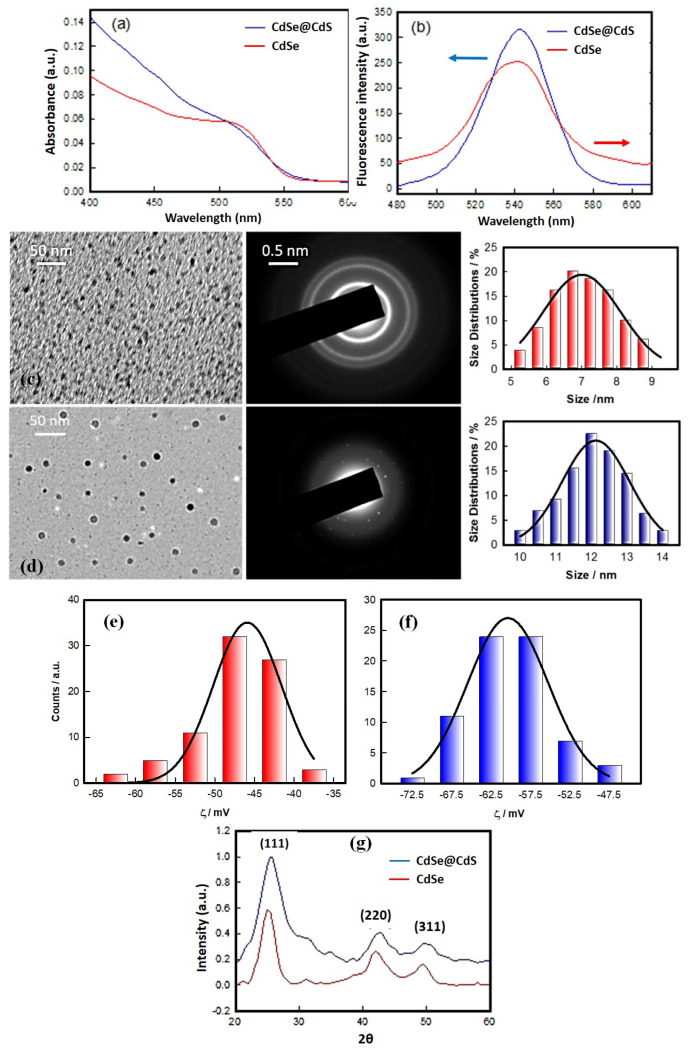
Characterization of CdSe and CdSe@CdS core-shell NCs. (**a**) UV-Vis absorption spectra; (**b**) fluorescence emission spectra (excitation wavelength ≈ 505 nm). (**c**,**d**) TEM images of CdSe (7 ± 1 nm) and CdSe@CdS core–shell (12 ± 1 nm) NCs, respectively. (The size histograms are shown on the right). (**e**,**f**) Zeta potential of CdSe (−45 ± 4) mV and CdSe@CdS core–shell (−60 ± 5) mV NCs, respectively. (**g**) X-ray diffraction patterns.

The physical size of the NCs was measured using DLS, TEM, and UV–Vis absorbance data (Figure 1). While DLS and TEM directly yielded the values for mean size, we used the empirical relation proposed by Jasieniak et al. to derive a size value from the UV–Vis spectral data. The empirical formula determined the particle diameters, D, of the NCs from their absorption spectra [38].

(3) $$D\;(nm)=59.60816-0.54736\;\lambda +1.8873\;\times \;10^{-3}\lambda^{2}-2.85743\;\times \;10^{-6}\lambda^{3}\;+1.62974\;\times \;10^{-9}\lambda^{4}$$

where D (nm) is the size of QD, and λ (nm) is the corresponding wavelength to the first excitonic absorption peak of the sample. It must be noted that the D values determined were 2.4 ± 1.0 and 5.9 ± 1.0 nm for CdSe and CdSe@CdS NCs, respectively. However, this is a gross underestimate of the particle size. The DLS method produced a larger size because of the hydration-mediated clustering of the NCs. Two observations could be readily made from the data presented in Table 1. These are (i) the core–shell structure was associated with a higher negative surface charge, and (ii) the thickness of the CdS layer on the CdSe core NC was on the order of 2.5 nm (Figure 1c,d).

The absorbance was measured for both the NCs; it showed a sharp peak at 505 ± 5 nm. One of the main objectives was to make the CdSe NCs fluorescent by cladding them with CdS. Fluorescence emission was measured by exciting both samples at 505 nm. The fluorescence emission peaks for the core–shell NCs were much higher than for the bare NCs. The presence of the CdS cladding was responsible for enhancing the fluorescence of the core CdSe nanoparticles. The crystallite sizes of the CdSe and CdSe@CdS nanocrystals was obtained using an X-ray diffractometer. The XRD patterns of the NCs are shown in Figure 1g and are indexed based on the cubic system. The observed inter-planar spacing determined from the respective prominent peaks in the diffractograms correspond to reflections arising from the (111), (220), and (311) planes of the CdSe NCs, which are consistent with JCPDS Card No. 32-0483. Thus, the NCs had face-centered cubic (fcc) structures with crystallite size, inter-planar spacing, lattice strain, and plane Miller indices, as depicted in Table 2. The inter-planar d-spacings were determined from the (111) reflection peak to be 3.54 Å and 3.52 Å for the CdSe and CdSe@CdS NCs, respectively.

### 3.2. Cytotoxicity Analysis for Biocompatibility Screening

**Figure 2:**
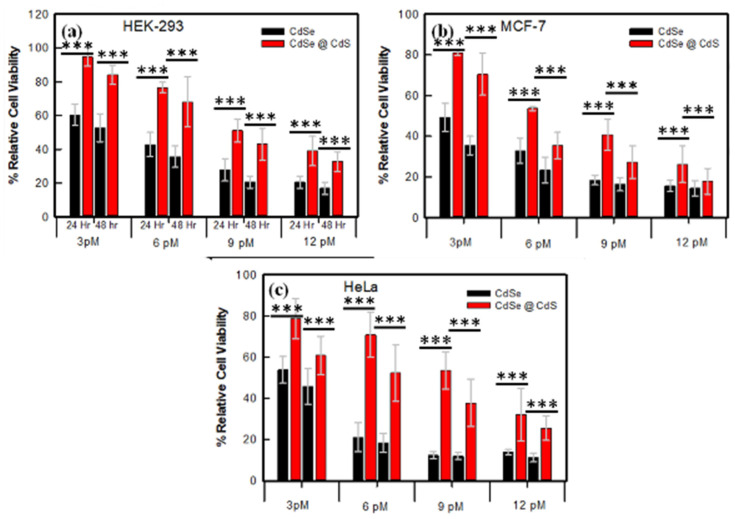
Comparative cell viability of CdSe and CdSe@CdS nanocrystals at 24 and 48 h assessed using the MTT assay. (**a**) Human kidney embryo (HEK-293), (**b**) human breast cancer (MCF-7), and (**c**) human epitheloid cervix carcinoma (HeLa). Statistical analysis of variation in data shown in figures by one-way ANOVA and Tukey’s test. *** *p* < 0.001 compared to control, or between the indicated groups.

The MTT assay is one of the simplest, fastest, and relatively cheap methods for screening cell viability. The viability of all studied cells (MCF-7, HEK-293, and HeLa) decreased as a function of time and dose for both CdSe and CdSe@CdS NCs, but the cytotoxicity rate was higher for CdSe-treated cells as compared to CdSe@CdS-treated cells. Meanwhile, the cancerous cell lines (MCF-7 and HeLa) treated with CdSe showed ∼85–90% cytotoxicity at a dose of 12 pM, but for HEK-293 cells it was ~70%, whereas in CdSe@CdS-treated cells the cytotoxicity rates were ~75, 70, and 55% in MCF-7, HeLa, and HEK-293 cells, respectively (Figure 2). It is evident that the cytotoxicity in the case of both CdSe and CdSe@CdS NCs was higher in cancerous cell lines compared to normal cells. Due to large membrane pore sizes, cancerous cells take up more nanoparticles which may be the cause of the observed cytotoxicity. We tested these nanoparticles’ efficacy in p53 HeLa, and p53 mutated breast cancer cell line MCF-7, which yielded different results. The LC_50_ values of CdSe nanoparticles in MCF-7 and HeLa were 3.09 and 2.79 pM, respectively, which were enhanced to 7.17 and 9.15 pM in CdSe@CdS-treated cells. In normal cells (HEK-293), the LC_50_ of CdSe@CdS nanoparticles was 10.02 pM, significantly higher than for CdSe-treated cells (4.5 pM). This indicates that surface modification reduced the toxicity of CdSe nanocrystals.

### 3.3. NC-Induced ROS Production

**Figure 3:**
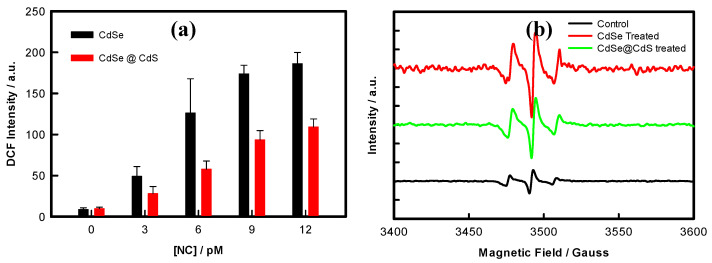
(**a**) ROS level in the HeLa cells after 12 h exposure to NCs at different concentrations, measured using DCFDA staining. (**b**) EPR spectra of HeLa cells after exposure to 12 pM NCs.

The DCF fluorescence, i.e., an indicator of oxidative stress (OS), was measured in the cells after 24 h treatment with CdSe and CdSe@CdS NCs. The reactive oxygen species level in the CdSe NC-treated cells was higher than in the CdSe@CdS NC-treated cells at the same concentration (Figure 3a). The fluorescence intensity of the DCF-positive cells increased significantly in a dose-dependent manner with CdSe NCs. In contrast, for the cells treated with CdSe@CdS NCs, the DCF intensity did not increase significantly. These results indicate that the surface coating of CdS reduced the release of free radicals, which may be the reason for the lower oxidative stress in the CdSe@CdS NC-treated cells.

### 3.4. Detection of Free Radicals by EPR

The generation of free radicals is the leading cause of nanomaterials-mediated toxicity; therefore, to detect and quantify the combined generation of superoxide and hydroxyl free radicals, the more sensitive EPR spectroscopy was used, using a DMPO spin trap. As depicted from the EPR spectra of the DMPO adducts, the CdSe NC-treated group resulted in six sharp peaks (peak nos. 1–3 in Figure 3b), with greater intensity as compared to the CdSe@CdS NCs and control peaks. The prominent middle peak in all three spectra represents a g value equal to 2.006 characteristics for the DMPO-OH adduct. The relative free radical concentration (combined concentrations of superoxide anions and hydroxyl free radicals) inside the HeLa cells can be measured by calculating the increase in the central peak intensity in the treated cells compared to the untreated cells. Increased free radicals in the CdSe NCs, with intensified peaks, clearly revealed the generation of more hydroxyl free radicals in the CdSe NCs group compared to the CdSe@CdS NCs and the control. These alterations were supported by ROS production measurements using DCFDA staining.

**Figure 4:**
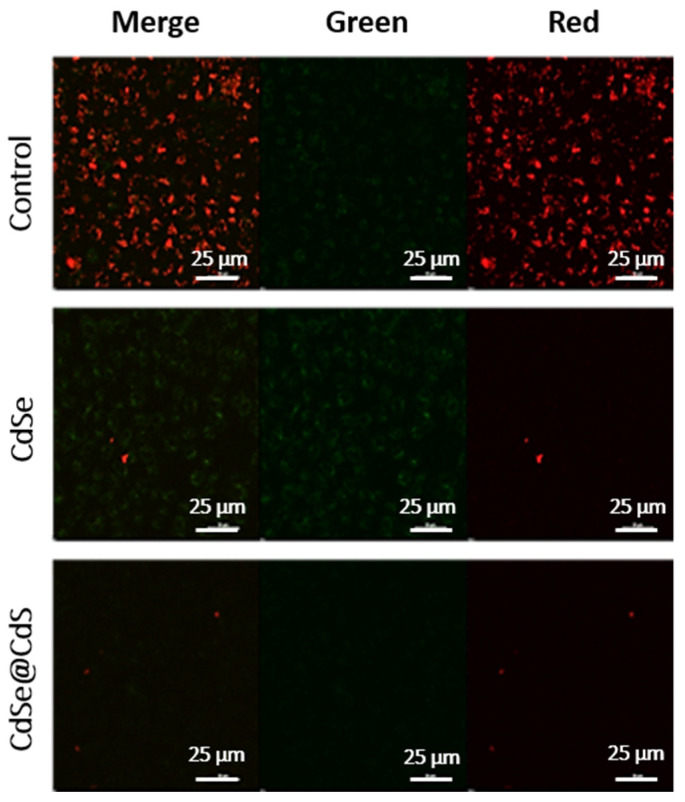
Mitochondrial membrane depolarization of HeLa cells exposed to 12 pM NCs for 12 h measured using JC-1 staining; the image was captured using a live imaging microscope (Nikon Eclipse Ti) at 20× magnification.

To study the effect of ROS in the mitochondria-mediated apoptosis pathway, the mitochondrial membrane potential (ΔΨm) was measured, because it regulates mitochondrial permeability and plays a prominent role in initiating apoptosis. One can easily observe in Figure 4 that there is a decrease in ΔΨm from 100% (untreated cells) to 55% and 35% in cells treated with 12 pM of CdSe and CdSe@CdS NCs, respectively. These results suggest that the mitochondrial pathway is possibly playing a substantial role in regulating the induction of apoptosis caused by these NCs. Still, the damage intensity was more significant in core-only than in core–shell NCs.

**Figure 5:**
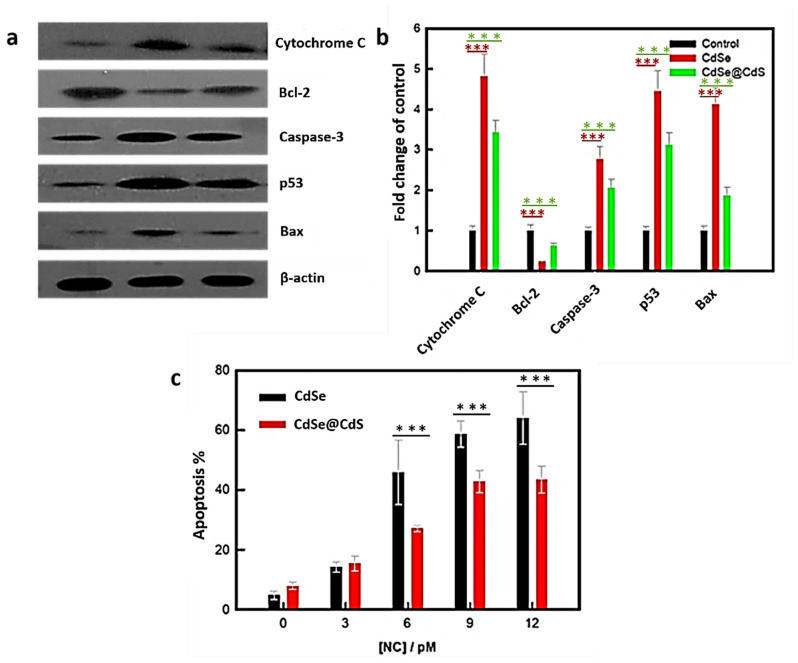
Effects of NCs of the molecular mechanism of apoptosis. (**a**) Changes in the expression of apoptosis regulatory proteins in response to treatment with 12 pM CdSe CdSe@CdS NCs. (**b**) Bar diagram represents the mean change in band intensity (protein level/b-actin value normalized with untreated control). (**c**) Apoptosis percentage of HeLa cells after 12 h exposure to different NCs (with and without CdS coating) at different concentrations. Statistical analysis of variation in data shown in (**b**,**c**) using one-way ANOVA and Tukey’s test, *** *p* < 0.001 compared to control, or between the indicated groups.

In the present study, core-only and core–shell NCs increased ROS production in HeLa cells dose dependently. To investigate whether these NCs can trigger an intrinsic apoptotic cascade, we examined the the expression of p53, Bax, Bcl-2, and caspase-3 proteins in the treated cells and control cells using western blot analysis. The results demonstrated that the expression of pro-apoptotic markers such as p53, Bax, and caspase-3 was significantly elevated in CdSe and CdSe@CdS NC-treated cells. At the same time, Bcl-2 decreased in both groups of NC-treated cells (Figure 5a,b). Meanwhile, the release of cytochrome c was found more in core-only than core–shell NC-treated cells. The intensity alteration was higher in CdSe-treated cells than in CdSe@CdS NC-treated cells. These results suggested that both the NCs induced apoptosis through the intrinsic pathway. CdSe and CdSe@CdS both increased apoptotic cell populations in a dose-dependent manner. There was a significant increase in the apoptosis rate in the CdSe-treated group compared to the control group (Figure 5c). The cells treated with lower concentrations of CdSe@CdS did not show any significant change in apoptosis compared to the control group. Still, higher concentrations of CdSe@CdS caused a substantial increase in cell apoptosis in a dose-dependent manner.

### 3.5. Effect on Cellular Morphology

**Figure 6:**
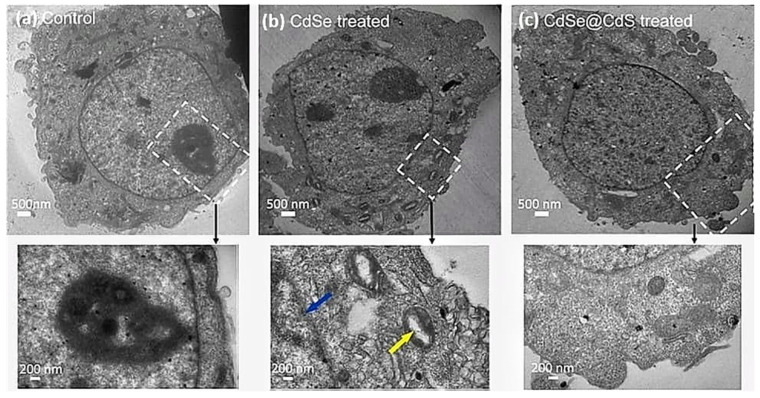
TEM images of HeLa cells exposed to different NCs (with and without CdS coating) for 24 h: (**a**) control HeLa cells without treatment, (**b**) HeLa cells treated with 6 pM CdSe, (**c**) HeLa cells treated with 6 pM CdSe@CdS. The blue arrow shows the deterioration of the nuclear membrane due to particle interaction. The yellow arrow indicates vacuolization in mitochondria.

The present study observed significant dose-dependent morphological changes characteristic of apoptosis. Cells were found to show apoptosis features, i.e., cell shrinkage in the CdSe-treated group, whereas CdSe@CdS did not induce any significant change in the cell integrity when applied at lower doses. However, changes were more significant in CdSe NC-treated cells than CdSe@CdS NC-treated cells at similar doses. The ultra-structural results show that the control cells were large and round, with an intact nuclear membrane and low density of nuclear chromatin. However, the cells treated with CdSe nanocrystals exhibited characteristics of apoptosis, including vacuolization in mitochondria, condensation, and fragmentation of nuclear chromatin adjacent to the nuclear membrane. In contrast, the cells treated with CdSe@CdS showed no significant alteration. These results show that CdSe@CdS caused no significant morphological damage, whereas CdSe nanocrystals induced cell apoptosis. This indicates that coating a CdS shell onto a core of CdSe reduced the release of free radicals, which may have caused cytotoxicity (Figure 6).

### 3.6. Cellular Uptake

**Figure 7:**
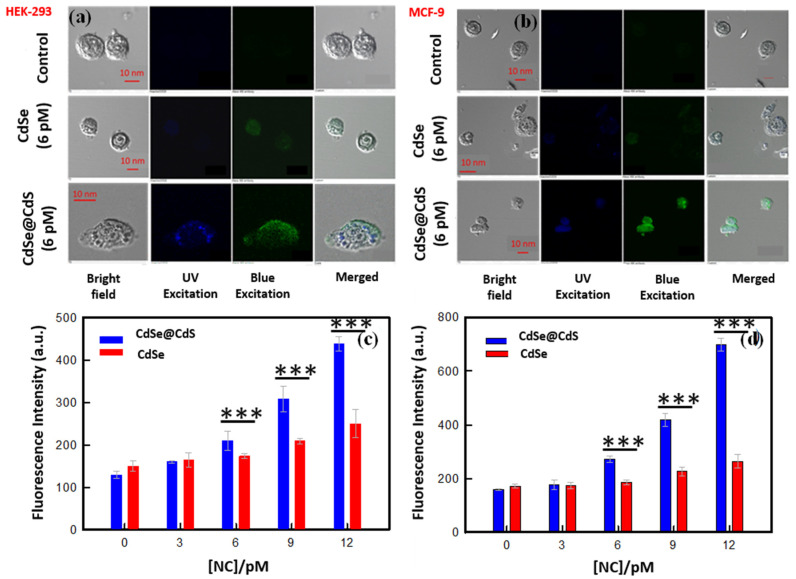
Localization of NCs in the (**a**) HEK-293 and (**b**) MCF-7 cells. In all three panels, the left side column represents the phase contrast image, while the central left column represents the fluorescence image using UV light excitation, and the central right column represents the fluorescence image using blue light excitation. The right column is an overlay of all columns. In all cases, NCs which are more localized inside the cells reveal finer structures. The histogram shows the fluorescence intensity of NCs inside the cells: (**c**) HEK-293 and (**d**) MCF-7. Statistical analysis of variation in data shown in (**c**,**d**) by one-way ANOVA and Tukey’s test. *** *p* < 0.001 compared to control, or between the indicated groups.

Confocal laser microscopy is a widely used technique for high-resolution imaging of cells. Fluorescent CdSe quantum dots are ideal probes for imaging cells [39]. Remarkably, we noticed that fluorescent CdSe nanocrystals were internalized by HeLa and HEK-293 cells and adsorbed onto the cell membrane. Still, the fluorescence intensity was low, at only two times the control. When these cells were treated with the same concentration (12 pM) of CdSe@CdS nanocrystals, the fluorescence intensity increased by roughly four times (Figure 7). This implies the cellular internalization of these nanocrystals. At higher dosages of CdSe nanocrystals, cell membranes were found to be ruptured due to the generation of reactive oxygen species. The comparative imaging revealed distinctive features of normal cell lines (HEK-293) and cancerous cell lines (HeLa). It was clearly observed that CdSe@CdS (and CdSe) adsorption was higher in HeLa cells compared to HEK-293 cells treated with the exact same dosage of nanocrystals. This difference in internalization between cancerous and normal cell lines could be assigned to different degrees of cell surface charge, the thickness of the membrane, and the higher turnover rate of cancer cells compared to normal cells. This study revealed that the enhanced fluorescence intensity and biocompatibility of the core–shell structures of CdSe@CdS demonstrate the potential to be used in cell imaging applications.

## 4. Discussion

The heavy metal cadmium (Cd) is highly toxic and harms several biological systems. Its capacity to accumulate in tissues and obstruct vital cellular functions gives it biological toxicity. Cd may enter the body after exposure by eating, inhalation, or cutaneous contact. Once absorbed, the kidneys, liver, lungs, and bones are its main targets [40]. There are several methods through which Cd poisoning might occur. Attaching to proteins and enzymes and causing their deactivation or structural modification interferes with cellular processes. Cd also produces reactive oxygen species (ROS), which cause oxidative stress and damage to proteins, lipids, and DNA in cells [40,41]. It can obstruct essential signaling pathways, compromise cell viability, and disturb calcium homeostasis. Cadmium exposure has a wide range of adverse effects. It is connected to damage to the kidney, which results in renal dysfunction and impairment of the filtration and reabsorption processes [41]. Cd poisoning also affects the pulmonary system, producing inflammation and lung fibrosis. It also affects bone metabolism, weakens bones, and increases fracture risk [40,41].

Several studies have reported the toxicity of CdSe particles against various cell lines. The potential toxicity induced by CdSe particles is basically due to the release of free Cd^2+^ ions into the medium that, in turn, interacts with the cells [30,31,32]. As there is a continuous increase in the use of semiconductor nanocrystals in biological and biomedical applications, there is a need to assess the cytotoxicity of these NCs prior to evaluating their application. Therefore, in this particular study, we assessed the cytotoxic behavior of CdSe particles against three different cell lines: HEK-293, MCF-7, and HeLa cell lines. We studied the cytotoxicity of a series of CdSe particles: a core CdSe quantum dot and a core–shell CdSe@CdS.

Physicochemical characterization studies have reported the high fluorescence and larger particle size of CdSe@CdS quantum dots compared to CdS particles. The variation in size is because of the presence of the CdS coating, which also provides an overall negative charge on the surface and correspondingly high stability. The cytotoxicity of these two particles against the HEK-293 (kidney), MCF-7 (breast cancer), and HeLa (cervical cancer) cell lines was determined. Viability data determined from the MTT assay was extrapolated in a dose-dependent curve, suggesting that the CdSe@CdS particles were less toxic than CdSe particles. Furthermore, we deduced the particles’ higher toxicity towards cancerous cell lines (HeLa and MCF-7) compared to the normal cell line (HEK-293). The probable reason behind the lower toxicity towards HEK-293 is the difference in the pore sizes of cancerous and normal cell lines. Cancerous cell lines have large membrane pore sizes which assists in a higher uptake of particles compared to normal cell lines, thus, inducing a higher toxic effect. The direct correlation between ROS generation and apoptosis is well known, and, therefore, we correlated the ROS activity in various cell lines after treatment with quantum dots. The CdS coating on the CdSe@CdS particles is responsible for the lower toxicity, as the surface coating, crystal size, and structure are known to be determining factors for cytotoxicity [30]. As expected, the high quantity of ROS generation in CdSe-treated cells compared to CdSe@CdS confirmed the distinctive in vitro cytotoxicity behavior on the basis of the difference in surface properties.

The endoplasmic reticulum (ER), peroxisomes, and especially the mitochondria are where reactive oxygen species (ROS) are largely produced by cells [42]. When molecular oxygen is used as a substrate for water synthesis during oxidative phosphorylation, the mitochondrial electron transport chain (ETC) introduces electrons into the reaction. A part of these electrons is taken up by molecular oxygen, forming superoxide (O_2_−), which can then go through additional processes to produce hydrogen peroxide (H_2_O_2_) and the hydroxyl radical (•OH) [43]. The generated ROS induce pore formation in mitochondria, resulting in mitochondrial permeability and deregulation of mitochondrial function [44]. Additionally, Bax and p53, the pro-apoptotic proteins, permeate the mitochondrial outer membrane and regulate apoptosis [45,46]. ROS are known to inhibit the anti-apoptotic protein Bcl-2 and assist in the activation and translocation of the pro-apoptotic protein Bax to the outer mitochondrial membrane (OMM), where it forms oligomers, which are important factors in the formation of permeability transition pores (PTP) and subsequent release of cytochrome c [47]. The release of cytochrome c into the cytosol via the mitochondrial pores disturbs the mitochondrial membrane potential by activating caspases and caspase-3 [48]. Activated caspase-3 is known to be the main executioner of apoptosis. In the present case, we observed mitochondrial membrane depolarization as well as elevated levels of caspase-3 on treatment with the nanocrystals, which demonstrated that the synthesized NCs induce cellular apoptosis via mitochondrial dysfunction and the caspase-3-mediated pathway. Another study reported that CdSe core nanocrystals induce cytotoxicity in IMR-32 human neuroblastoma cells via apoptotic biochemical changes, loss in mitochondrial membrane potential, and release of cytochrome c [16].

**Figure 8:**
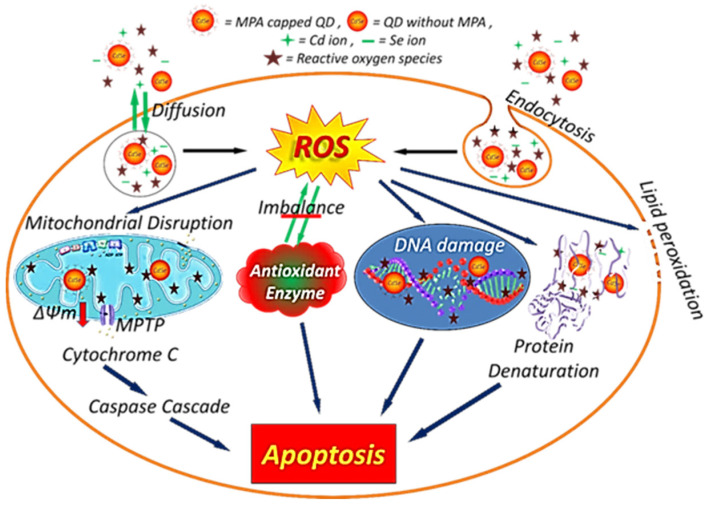
Schematic overview of the possible mechanisms of cellular toxicity by different pathways by CdSe and CdSe@CdS NCs.

Intracellular uptake of nanocrystals and their cellular localization is essential for inducing cell toxicity. We, therefore, identified the localization of these Cd-based NCs via confocal laser microscopy imaging that confirmed the uptake of both CdSe and CdSe@CdS NCs by the cells. The fluorescence intensity of the CdSe@CdS-treated cells was higher than the CdSe-treated cells and almost four times that of the control cells, which can be attributed to the difference observed during the physicochemical characterization. Thus, the low toxicity and high fluorescence property of CdSe@CdS nanocrystals makes them suitable agents for bioimaging and cellular tracking studies. Additionally, a higher uptake of CdSe@CdS NCs was seen in normal cells, which can be correlated with the distinctive structure and functional ability of cancerous cell lines compared to normal cell lines. Furthermore, we studied the nanocrystals’ subcellular distribution in HeLa cell lines and imaged it via high-resolution TEM analysis. The nanocrystals were internalized indiscriminately in all sizes, and a non-uniform distribution was observed. A higher uptake was seen for CdSe@CdS particles compared to CdS particles. The cellular uptake depends on the dose, time period of incubation, and the process of internalization. Distinctive destruction of the nuclear membrane was seen in CdSe-treated cells, which was induced via particle interactions. The nanocrystals initially adhere to the cell membrane, entering the cells via endocytosis and distributing across the cytosol. Smaller particles make their way to the cell nuclei, inducing nuclear damage [49]. Table 3 shows the reported Cd-based nano-architectonics used in cancer diagnosis. A schematic overview of the probable mechanism of cellular toxicity and apoptosis is depicted in Figure 8.

As the shape, size, surface modification, surface charge, and cellular morphology of particles are the major determining factors for cellular uptake and the corresponding toxicity, it is difficult to determine the systematic means of cytotoxicity. We inferred that the presence of a CdS coating improves the uptake of nanocrystals and makes the particles less toxic, enabling their use in biological applications. Our results, thus, suggested that the chemical composition and surface coating significantly determine the corresponding toxicity.

## 5. Conclusions

We investigated the effects of core (CdSe) and core–shell (CdSe@CdS) nanocrystals (NCs) on diverse cell lines in this work methodically and thoroughly. We considered various factors, such as cellular uptake and dispersion, cytotoxicity, and apoptosis. Notably, we saw various NC kinds behave in different ways. Depending on the NCs’ composition, different cells absorbed and distributed the NCs differently. When exposed to additional cell lines, core–shell structures showed lower cytotoxicity than core NCs. This research implies that a shell layer can reduce the negative impacts of the core NCs. Furthermore, we found that all cell lines effectively phagocytized the larger-sized core–shell NCs, as seen by the increased cellular uptake. This increased absorption is a sign of core–shell NCs’ lower cytotoxicity as compared to core NCs. Our research showcases the unprecedented significance of even the subtlest disparities in particle size and the precise selection of cell lines on the intricate mechanisms governing the uptake of these nanoscale clusters by cells. The study’s cellular imaging results demonstrate the interaction between CdSe NCs and cells and emphasize the potential of these molecules as anticancer agents. Further research is necessary to understand the underlying processes better, to maximize the potential use of NCs in biological applications, considering the intricacy of these interactions and the reported variances in cellular response.

## Figures and Tables

**Table 1:** Physical characteristics of the NCs.

NCs	Absorbance Maxima (nm)	Emission Maxima (nm)	TEM Size (nm)	Average Crystalline Size (nm)	DLS Size (nm)	Zeta Potential (mV)
CdSe	505	548	7 ± 1	3.10 ± 0.5	51 ± 5	−45 ± 4
CdSe_CdS	500	542	12 ± 1	2.70 ± 0.5	60 ± 7	−60 ± 5

**Table 2:** Variations in attributes of CdSe and CdSe@CdS NCs calculated from XRD analysis.

NC	Plane (hkl)	2θ (°)	FWHM (°)	Relative Intensity	Inter-Planar Spacing (Å)	Lattice Vector (Å)	Lattice Strain	Crystallite Size (nm)	Average Size (nm)
CdSe	(111)	25.2	3.83	0.579	3.54	6.13	0.0553	3.01	3.10
	(230)	42.1	3.00	0.253	2.15	6.08	0.0340	2.96	
	(311)	49.4	2.75	0.154	1.84	6.10	0.0261	3.32	
CdSe @CdS	(111)	25.3	3.86	0.996	3.52	6.10	0.0751	2.20	2.73
	(230)	42.3	3.04	0.404	2.14	6.05	0.0343	2.93	
	(311)	49.3	3.00	0.320	1.83	6.07	0.0283	3.05	

**Table 3:** Reported Cd-based nano-architectonics used in cancer diagnosis [50].

Cd-Based Nano-Architechtonics	Cell Line Used	Targeted Receptor
Polymer-coated CdSe/ZnS	Human oral squamous carcinoma cells (BcaCDE885)	Integrin αvβ3
PEGylated CdTe	Human glioblastoma cells (U87 MG cells)	Integrin αvβ3
Magnetic and CdTe QDs immobilized on SiO_2_	MCF-7 cells	Mucin 1 protein
CdTe QDs	MCF-7 cells	MMP-2
Mercapto-succinic acid-coated CdTe QDs	Fibroadenoma and ductal carcinoma	Glycans
CdTe QDs and Fe_3_O_4_ NPs;	Colon carcinoma cells LS174	TAG-72
CdTe/MPA QDs	Gastric cancer cell line MGC80-3	TAG-72
NAC capped and alloyed with CdTeS	Bel-7402 human hepatoma cells	Folate receptor
CdSe/ZnS coated with oleylamine poly (aspartate)-graft-PEGdodecylamine	Human liver cancer (HepG2) cells	VEGFR

## Data Availability

The data that support the findings of this study are available from first and the corresponding author upon reasonable request.
